# NKD2 as a Mediator of IFIX Antioncogene‐Induced Wnt Signalling and Epithelial–Mesenchymal Transition in Human OSCC

**DOI:** 10.1111/jcmm.70342

**Published:** 2025-01-20

**Authors:** Shan Wang, Haixia Fan, Jie Bai

**Affiliations:** ^1^ Department of Oral Pathology, School of Stomatology Hainan Medical University Haikou P. R. China; ^2^ Department of Oral Medicine Jining Medical College Jining P. R. China; ^3^ Department of Ophthalmology, the Fourth Affiliated Hospital Zhejiang University School of Medicine Yiwu P. R. China

**Keywords:** EMT, IFIX, NKD2, OSCC, Wnt

## Abstract

The activation of the human interferon‐inducible protein X (IFIX) isoform is associated with maintaining a stable cytoskeleton and inhibiting epithelial–mesenchymal transition (EMT). However, the mechanisms and pathways underlying IFIX‐mediated oncogenesis are not well understood. In this study, we investigated the effects of IFIX overexpression and knockdown in CAL‐27 and SCC‐25 oral squamous cell carcinoma (OSCC) cells. We observed significant variations in the expression of E‐cadherin, N‐cadherin, vimentin and Snail, as well as changes in wingless/integrated (Wnt) signalling. Our results indicated a strong correlation between IFIX and EMT, as evidenced by quantitative reverse‐transcription PCR and Western blotting, which revealed that Wnt3a and Wnt4 pathway components were regulated in IFIX‐overexpressing or knockdown cells, with naked cuticle 2 (NKD2) showing the strongest positive correlation. Both IFIX overexpression and knockdown modulated NKD2 expression. NKD2 silencing mimicked the phenotypic effects of IFIX knockdown, inhibiting E‐cadherin expression and increasing N‐cadherin, Snail and vimentin expression. Additionally, silencing NKD2 restored the anticarcinogenic phenotype associated with IFIX overexpression, affecting cell proliferation, invasion and migration. These findings provide mechanistic insights into the antioncogenic effects of IFIX in OSCC, involving the inhibition of Wnt signalling through NKD2, which leads to cancer‐inhibiting phenotypic effects, including restricted EMT.

**Abbreviations**
EMT: Epithelial–mesenchymal transition
FAK: Focal adhesion kinase
IFIX: Interferon‐inducible protein X
LRP: lipoprotein receptor
NKD2: Naked cuticle (Nkd) protein 2
OSCC: Oral squamous cell carcinoma
PCP: Planar cell polarity
POP: Processing of precursor
qRT‐PCR: quantitative reverse‐transcription PCR
ROR1/ROR2: Receptor tyrosine kinase‐like orphan receptors 1 and 2
RYK: Receptor‐like tyrosine kinase
TCGA: The Cancer Genome Atlas
Wnt: Wingless/integrated

## 1 Introduction

The human interferon‐inducible protein X alpha 2 (IFIX) isoform, which belongs to the interferon‐inducible HIN‐200 family, has been identified as a putative tumour suppressor. We previously showed that IFIX acts as a tumour suppressor and exerts positive effects by suppressing oral squamous cell carcinoma (OSCC). OSCC is a leading cause of maxillofacial malignant tumours, with 177,757 related deaths worldwide in 2020 [1]. The biological behaviour of IFIX involves cytoskeleton stabilisation via components such as cytokeratin and paxillin downregulation and is consequently associated with the epithelial–mesenchymal transition (EMT) in OSCC [2].

EMT is a dynamic process in which the migratory capacity and invasiveness of epithelial cells are enhanced by the loss of intercellular adhesion and polarity [3]. EMT is characterised by the acquisition of a mesenchymal phenotype by cells as they lose epithelial features [3]. The molecular mechanisms underlying EMT and IFIX‐mediated tumour suppression in OSCC are poorly understood.

Wingless/integrated (Wnt) signalling is a central mechanism involved in the regulation of tissue morphogenesis during embryogenesis and repair. The pivotal molecule of this signalling cascade is the Wnt ligand, which binds to receptors belonging to the Frizzled family or the receptor tyrosine kinase‐like orphan receptors 1 and 2 (ROR1/ROR2) and receptor‐like tyrosine kinase (RYK) families. This interaction governs the downstream canonical and noncanonical signalling cascades, such as Wnt processing of precursor (POP), which ultimately affects the cellular cytoskeleton, transcriptional control of proliferation and differentiation, and organelle dynamics, and consequently promotes the maintenance of EMT [4, 5, 6, 7, 8]. Anomalous Wnt signalling is associated with several malignancies, most prominently colorectal, breast, lung, oral, cervical and haematopoietic cancers [4, 5, 6, 7, 8].

Naked cuticle protein 2 (NKD2) is a 451‐amino‐acid protein. The primary functions of NKD2 are to suppress the Wnt/β‐catenin and Wnt/planar cell polarity (PCP) pathways [6, 9]. KKD2 can physically associate with DISHEVELLED 1 (homologous to Drosophila Dsh) (DVL1) and promote its degradation via K48‐linked polyubiquitylation, lowering cellular β‐catenin levels and attenuating the Wnt/β‐catenin pathway [6, 9]. NKD2 plays key role in the development of many cancer types, such as breast cancer, hepatocellular carcinoma, oesophageal cancer and osteosarcoma [10, 11, 12, 13]. However, whether NKD2 contributes to OSCC development and progression remains unclear.

Here, we hypothesise that IFIX exerts its tumour‐suppressive effects in OSCC by regulating NKD2 and the Wnt signalling pathway, which in turn affects EMT and cell adhesion. To test this hypothesis, we examined the function of the IFIX antioncogene by knockdown or overexpression of IFIX in OSCC cell lines and then pathologically analysed IFIX‐positive clinical oral cancer specimens. We found that IFIX upregulated E‐cadherin expression and inhibited Wnt signalling. Silencing NKD2 mimicked the phenotypic effects of IFIX knockdown, and NKD2 overexpression restored the phenotypic effects of IFIX knockdown. These findings indicate a link between IFIX activation in vitro and activation of the Wnt pathway through NKD2, which results in EMT and changes in cell adhesion. Data about IFIX in oral cancer from The Cancer Genome Atlas (TCGA) support the notion that IFIX expression plays an important role in inhibiting nodal metastasis.

## 2 Materials and Methods

### 2.1 Cell Culture and Infection

Cell culture and IFIX overexpression infection were as previously described [2]. IFIX‐ shRNA, NKD2‐, NC shRNA lentiviral vectors or NC plasmids were constructed by GENECHEM Biotech in Shanghai, China (http://genechem.bioon.com.cn/). Transfection procedures were performed according to provided protocols. Briefly, cells (1 × 10^4^ per well) grown in 24‐well plates were transduced with lentiviruses for 24 h, then cells were selected with puromycin (4 μg/mL) 48 h posttransduction for 6–10 days and expanded. Overexpression or knockdown of IFIX and NKD2 was assessed by quantitative reverse‐transcription PCR (qRT‐PCR) and Western blot. Empty vector was performed using Lipofectamine 3000 (Life Technologies) with 10 μg plasmid DNA. Cells were incubated for 48 or 72 h after transfection before testing for transgene expression or performing downstream experiments.

### 2.2 Apoptosis Assays, Wounding Healing Assay and Invasion Assay

Those were as previously described [2].

### 2.3 CCK‐8

Cell proliferation was detected by CCK‐8. Cells (1000 per well) were cultivated on 96‐well plates and cell proliferation was detected for 96 h with cell counting kit‐8 (Beyotime, Shanghai, China). The optical density (OD) values were then measured at 450 nm.

### 2.4 Wounding Healing Assay

This was previously described [2]. Briefly, vector, OE‐CAL‐27, NC + NC, sh‐NKD2 and OE‐IFIX‐shNKD2 were plated in 96‐well culture dishes, serum‐starved for 12–16 h, and wounded with a 96 Wounding Replicator. The culture dishes were washed three times with 0.5% FBS to remove detached cells, and the remaining cells were grown in DMEM containing 10% FBS. After 8 and 24 h of incubation, migration was quantified by counting the cells (Geligo) that had moved beyond the reference line.

### 2.5 Invasion Assay

In vitro invasion assay experiments were conducted using Corning BioCoat Matrigel Invasion Chambers (Corning, NY, USA). These were as previously described [2].

### 2.6 The In Vivo Tumorigenesis Assay

All animal studies in this study were approved by the Committee on Use and Care of Animals at the Hainan Medical University, and conducted in accordance with their guidelines. Four‐week‐old female BALB/c nude mice were purchased from SPF (Beijing, China) and randomly divided into groups (*n* = 4). SCC‐25 cells were injected into the back region of mice. Tumour size and body weight of the mice were measured every 3 days. Tumour volumes were calculated according to the following equation: volume = length × width (mm)^2^/2. At the conclusion of the experiment, tumours were harvested and weighed.

### 2.7 Quantitative Real‐Time PCR

Total RNA was extracted according to the manufacturer's protocol for TRIzol Reagent (Invitrogen, 15,596,026). cDNA was prepared using a cDNA reverse‐transcription kit (Transgene Biotech, AT311‐03). Quantitative RT‐PCR was performed by using iTaq Universal SYBR Green Supermix (Bio‐Rad, 1,725,125) in a CFX96TM Real‐Time System (Bio‐Rad, Hercules, CA, USA). The reactions were performed in triplicate. The detailed primer sequences, including their length, Tm, and genomic positions, are provided in Table 1 for reference.

**TABLE 1:** Primer information.

Gene	Primer Type	Sequence	Length (bp)	Tm (°C)	Position
IFIX	Forward Primer	CCAAGCAACCGTCTCACAG	19	60.7	361–379
IFIX	Reverse Primer	GCCGAGTCTGCTCTTTGGA	19	61.7	481–463
Βactin	Forward Primer	CATGTACGTTGCTATCCAGGC	21	60.8	393–413
Βactin	Reverse Primer	CTCCTTAATGTCACGCACGAT	21	60.2	642–622
WNT3A	Forward Primer	AGCTACCCGATCTGGTGGTC	21	62.9	55–74
WNT3A	Reverse Primer	CAAACTCGATGTCCTCGCTAC	20	60.5	484–464
WNT4	Forward Primer	GTACGCCATCTCTTCGGCAG	20	62.7	336–355
WNT4	Reverse Primer	GCGATGTTGTCAGAGCATCCT	21	62.5	479–459
NKD2	Forward Primer	AGCGCAGATGACGGAGAGA	19	62.7	256–274
NKD2	Reverse Primer	CGAGACATCGCACTGGAGT	19	61.4	351–333

### 2.8 Western Blot Analysis

Western blot analysis was performed as previously described [2], with the following detailed steps. Total protein was extracted using RIPA buffer supplemented with protease and phosphatase inhibitors (Thermo Fisher Scientific, USA). Protein concentrations were determined using the BCA Protein Assay Kit (Thermo Fisher Scientific, 23,227). Equal amounts of protein (30 μg) were separated on 10% SDS‐PAGE gels and transferred to PVDF membranes (MilliporeSigma, IPVH00010). Membranes were blocked in 5% nonfat milk in TBST (Tris‐buffered saline containing 0.1% Tween‐20) for 1 h at room temperature.

The membranes were probed with the primary antibodies listed in Table 2, which provides detailed information including antibody names, sources, catalog numbers, and working dilutions:

**TABLE 2:** Antibody information in WB.

Antibody Name	Catalogue Number	Source	Dilution
Anti‐IFIX	NBP1‐79436	Bio‐Techne	1:1000
Anti‐E‐cadherin	20,874‐1‐AP	Proteintech Group	1:20000
Anti‐N‐cadherin	22,018‐1‐AP	Proteintech Group	1:20000
Anti‐Snail	ab216347	Abcam	1:1000
Anti‐vimentin	ab92547	Abcam	1:500
Anti‐NKD2	16,699‐1‐AP	Proteintech	1:1000
Anti‐Wnt3a	sc‐136,163	Santa Cruz Biotechnology	1:200
Anti‐Wnt4	sc‐376,279	Santa Cruz Biotechnology	1:200
Anti‐β‐catenin	ab32572	Abcam	1:1000
Anti‐GAPDH	G8795	MilliporeSigma	1:5000

Membranes were incubated with primary antibodies overnight at 4°C. After washing three times with TBST (10 min each), the membranes were incubated with HRP‐conjugated secondary antibodies (1:5000, Invitrogen, USA) for 1 h at room temperature. Immunoreactive bands were visualised using the ECL Western Blotting Substrate (GE Healthcare, RPN2232), and chemiluminescent signals were captured with the ChemiDoc Imaging System (Bio‐Rad, Hercules, CA, USA). Band intensities were quantified using Gel‐Pro Analyser 4.0 software (Media Cybernetics, Rockville, MD, USA) and normalised to GAPDH as the internal control. Each experiment was performed in triplicate, and representative images were presented. Membranes were cut before antibody incubation to detect proteins of different molecular weights, ensuring specificity and avoiding signal overlap.

### 2.9 Immunohistochemistry (IHC)

IHC was performed to evaluate the expression of NKD2, E‐cadherin, N‐cadherin, vimentin and Snail in tumour tissues. The paraffin‐embedded tumour tissues were sectioned at a thickness of 4 μm, deparaffinised in xylene and rehydrated through graded alcohols. Antigen retrieval was conducted using citrate buffer (pH 6.0) in a microwave for 15 min. After cooling to room temperature, the sections were treated with 3% hydrogen peroxide for 10 min to block endogenous peroxidase activity, followed by blocking with 5% bovine serum albumin (BSA) for 30 min. Primary antibodies were applied to the sections and incubated overnight at 4°C. The following antibodies were used in this study. Detailed information on the antibodies, including their sources and dilutions, is provided in Table 3.

**TABLE 3:** Antibody information in IHC.

Antibody Name	Catalogue Number	Source	Dilution
Anti‐NKD2	bs‐1926R	Bioss	1:200
Anti‐E‐cadherin	14472S	CST	1:300
Anti‐N‐cadherin	Ab76011	Abcam	1:500
Anti‐vimentin	ab92547	Abcam	1:400
Anti‐Snail	13099‐1‐AP	Proteintech	1:200

*Note:* The slides were washed three times with PBS, followed by incubation with a biotinylated secondary antibody at room temperature for 30 min. Signal detection was performed using a DAB substrate kit, and the sections were counterstained with haematoxylin. After dehydration and mounting, the slides were imaged using a light microscope (model). Representative images were acquired at 20× magnifications.

### 2.10 Statistical Analysis

Two‐group comparisons were made using a two‐tailed Student's t‐test; three or more different groups were evaluated by one‐way ANOVA followed by Bonferroni's post hoc comparisons. Statistical significance was set at *p* < 0.05. Statistical analyses of the data were performed using GraphPad Prism 6.

## 3 Results

### 3.1 Validation of IFIX Knockdown and Overexpression in CAL‐27 Cells and SCC‐25 Cell

**FIGURE 1:**
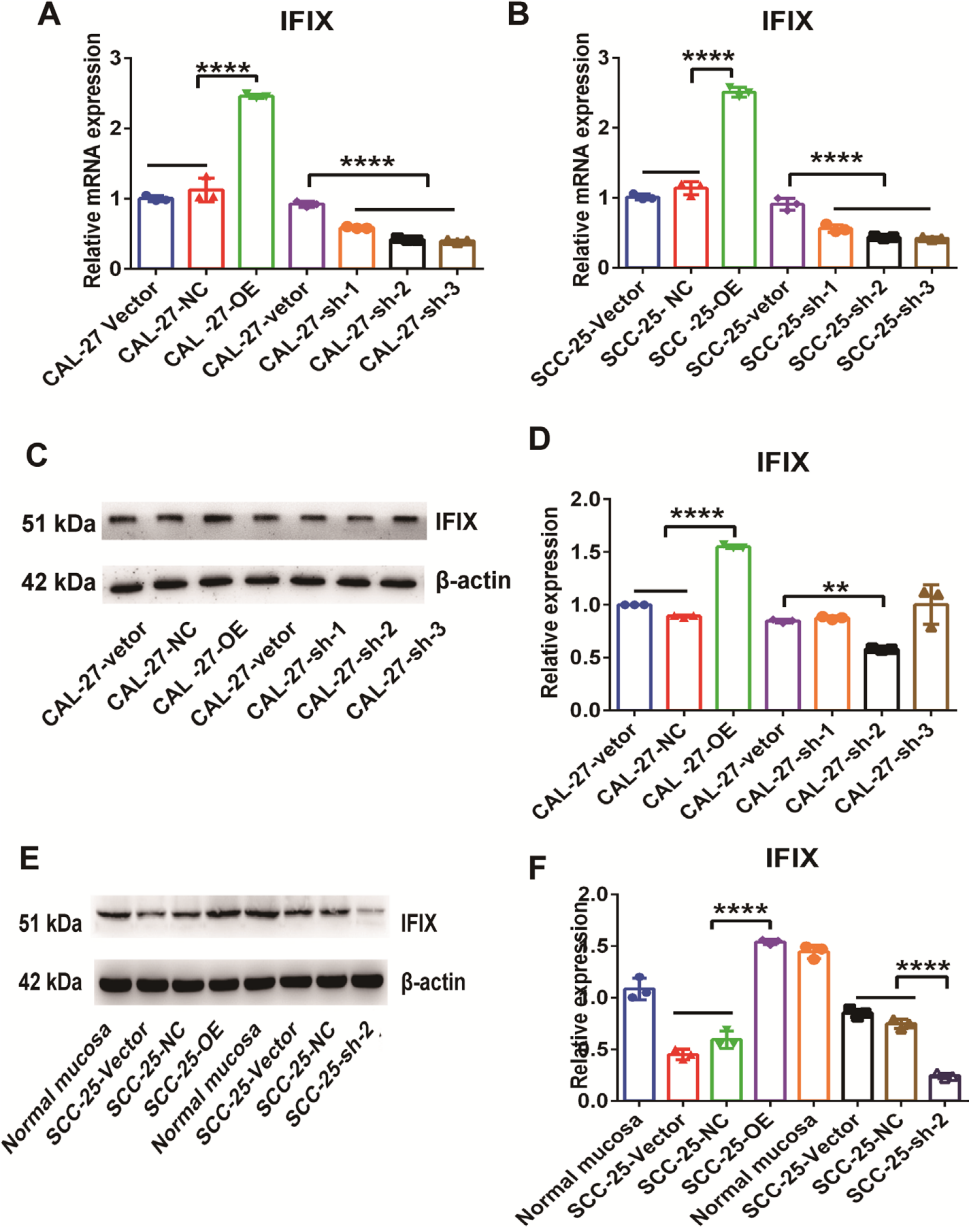
Validation of knockdown and overexpression of IFIX in CAL‐27 and SCC‐25 cells. (A,B) qRT‐PCR analysis results showing relative IFIX mRNA expression levels in CAL‐27 and SCC‐25 cell lines. (C–F) Western blot analysis and its quantification showing relative IFIX protein expression levels in CAL‐27 and SCC‐25 cells stably expressing shRNA molecules. Each qRT‐PCR and Western blot experiment was repeated three times, and data are presented as mean ± standard error (SE), ****p < 0.0001**p < 0.01.

Permanent IFIX knockdown in CAL‐27 and SCC‐25 cells was induced using short hairpin RNA (shRNA) lentiviral constructs. The results of qRT‐PCR (Figure 1A,B) demonstrated a significant upregulation of IFIX mRNA in CAL‐27 and SCC‐25 cells overexpressing IFIX (OE‐IFIX) and a notable downregulation in shRNA‐treated cells (sh‐IFIX). Western blotting indicated a 40%–60% knockdown compared with that in the shRNA control. The reduction in IFIX mRNA levels was reasonable in the IFIX shRNA2 and shRNA3 cell lines. ShRNA2 is identified in cells (Figure 1C–F). This cell line was subsequently used for functional studies. The overexpression findings were similar to our previous results [2]. These findings indicate the successful construction of stable IFIX knockdown cell lines, providing a reliable model for subsequent functional studies.

### 3.2 Inhibitions of EMT Variations by IFIX in OSCC Cells

**FIGURE 2:**
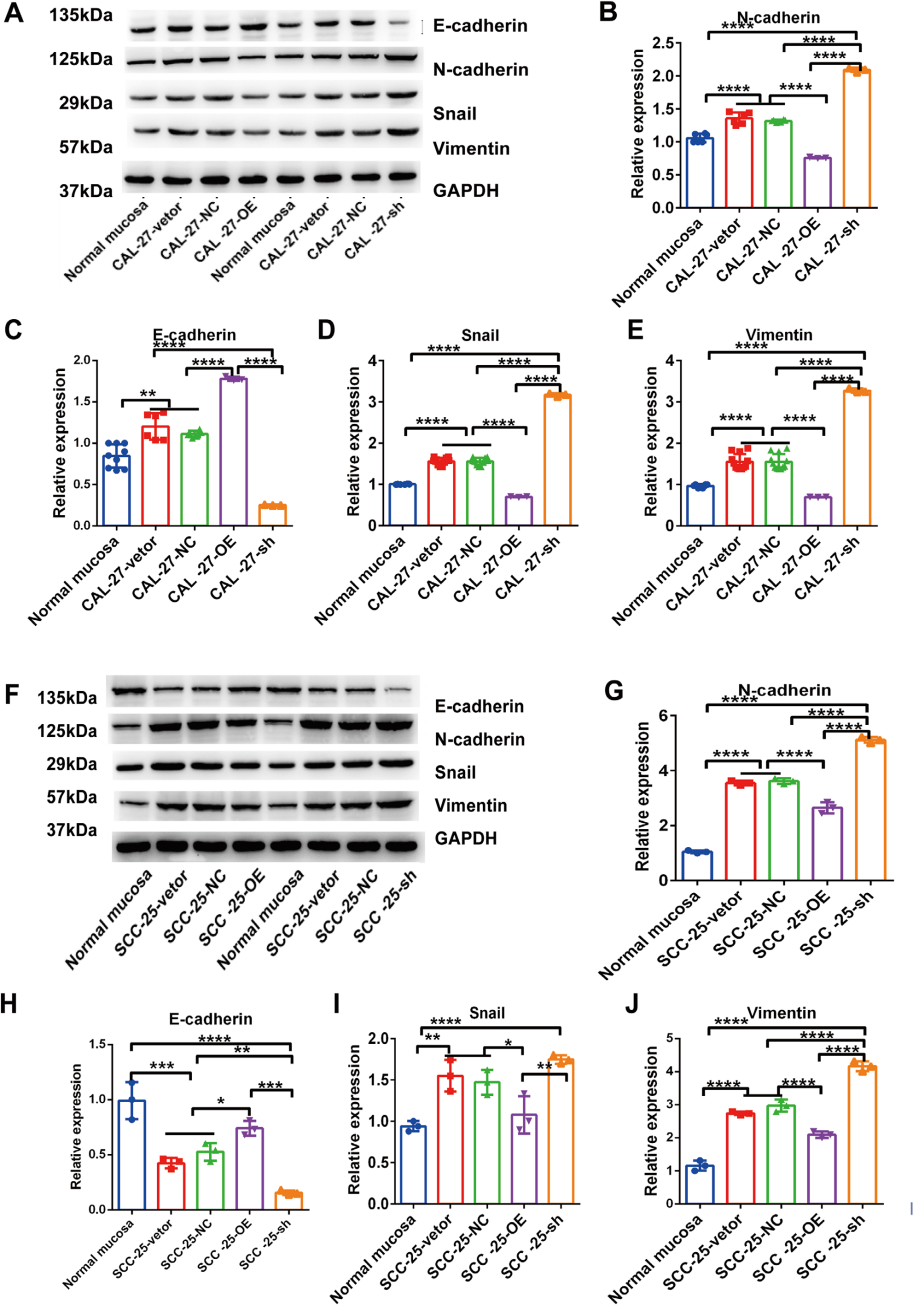
Inhibition of EMT by IFIX in OSCC cells. (A) Western blot analysis showing the expression levels of EMT phenotype markers (N‐cadherin, E‐cadherin, Snail and vimentin) in different treatment groups: Normal mucosa, CAL‐27‐vector, CAL‐27 cells (CAL‐27NC), overexpressed IFIX CAL‐27 cells (CAL‐27‐OE) and sh‐IFIX CAL‐27 cells (CAL‐27‐sh). GAPDH was used as a loading control. (B‐E) Quantification of Western blot results for N‐cadherin, E‐cadherin, Snail and vimentin expression levels. Error bars indicate mean ± SD. *p < 0.05, **p < 0.01, ***p < 0.001. (E) Western blot analysis of EMT markers in SCC‐25 cells treated with the same groups as in (A). GAPDH was used as a loading control. (F–J) Quantification of Western blot results for N‐cadherin, E‐cadherin, Snail and vimentin expression levels. Error bars indicate mean ± SD. *p < 0.05, **p < 0.01, ***p < 0.001, ****p < 0.0001.

We investigated phenotypic changes in CAL‐27 and SCC‐25 OSCC cells in response to IFIX knockdown and overexpression, utilising stable shRNA or transient small interfering RNA (shRNA) transfection methods. We measured the expression levels of classic epithelial (E‐cadherin) and mesenchymal (N‐cadherin, vimentin and Snail) markers during IFIX overexpression and knockdown to explore the relationship between IFIX and EMT. Overexpression of IFIX resulted in the upregulation of E‐cadherin and the downregulation of N‐cadherin, vimentin and Snail in CAL‐27 cells (Figure 2A–E), as well as in SCC‐25 cells transfected with shIFIX (Figure 2F–J), compared to control cells. These findings indicate that IFIX can inhibit EMT in OSCC cells.

### 3.3 IFIX Inhibits Cell‐Autonomous Wnt Signalling and Promotes NKD2 Expression in OSCC Cells

**FIGURE 3:**
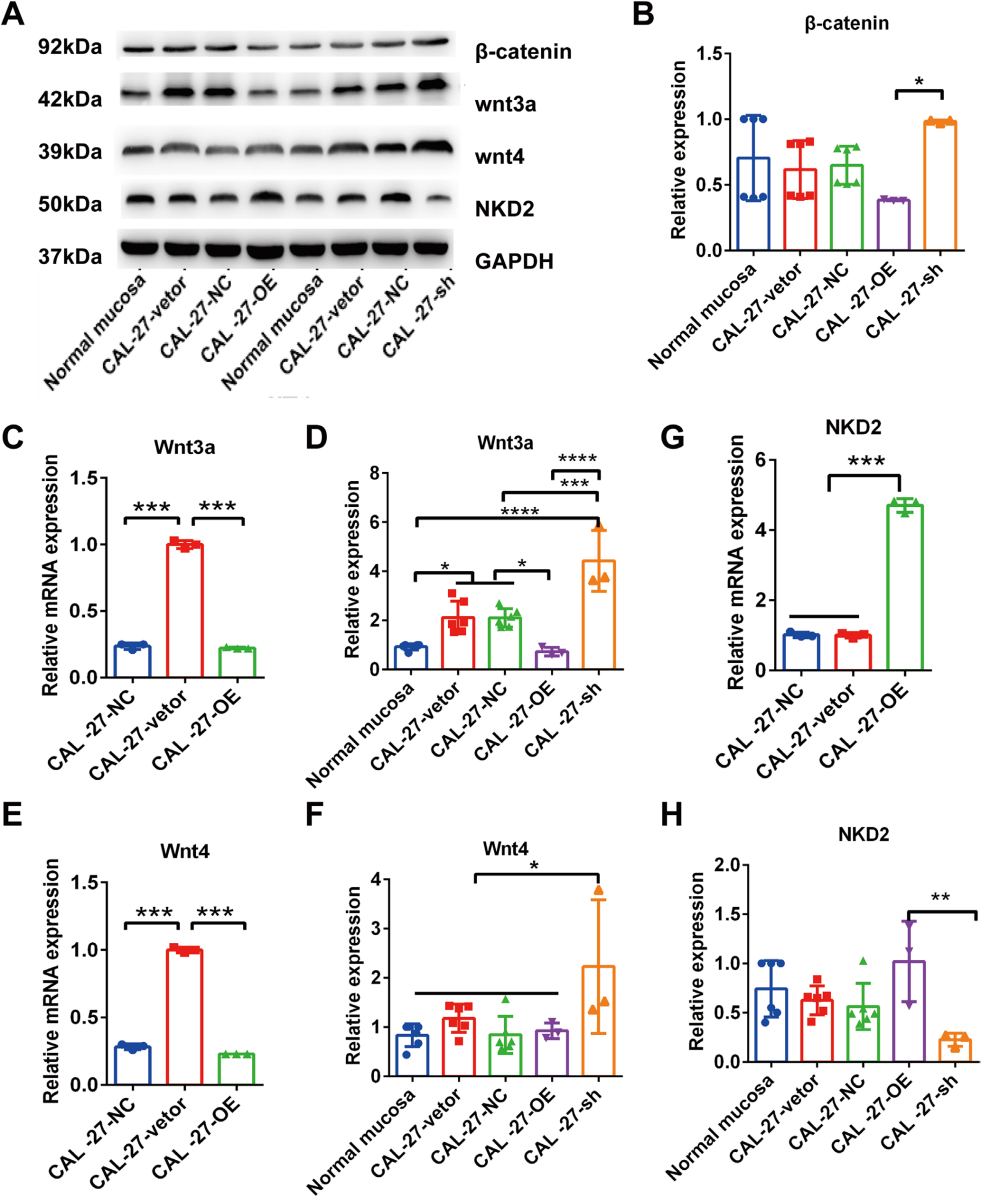
IFIX regulates Wnt signalling members including Wnt3a, Wnt4 and NKD2. (A) Western blot analysis of cell‐autonomous Wnt signalling after overexpression and knockdown of IFIX. (B) Quantification of β‐catenin expression by Western blot analysis in the CAL‐27‐vector, CAL‐27 cells (CAL‐27NC), overexpressed IFIX CAL‐27 cells (CAL‐27‐OE) and sh‐IFIX‐CAL‐27‐cells (CAL‐27‐sh). (C–F) Wnt3a and Wnt4 expression by qRT‐PCR analysis in CAL‐27 cells (CAL‐27NC), overexpressed IFIX CAL‐27 cells (CAL‐27‐OE) and Western blot analysis in CAL‐27‐vector, CAL‐27 cells (CAL‐27NC), overexpressed IFIX CAL‐27 cells (CAL‐27‐OE) and sh‐IFIX‐CAL‐27 cells (CAL‐27‐sh). (G‐H) NKD2 expression by qRT‐PCR analysis in CAL‐27 cells (CAL‐27NC), overexpressed IFIX CAL‐27 cells (CAL‐27‐OE) and Western blot analysis in the CAL‐27‐vector, CAL‐27 cells (CAL‐27NC), overexpressed IFIX CAL‐27 cells (CAL‐27‐OE) and sh‐IFIX‐CAL‐27 cells (CAL‐27‐sh). GAPDH was used as loading control. Error bars indicate mean ± SD. *p < 0.05, **p < 0.01, ***p < 0.001, ****p < 0.0001.

To investigate the mechanism by which Wnt signalling regulates the EMT process, we used Western blotting to examine β‐catenin regulation in CAL‐27 cells with IFIX overexpression or knockdown. The results showed that IFIX overexpression led to a significant downregulation of β‐catenin in CAL‐27 cells compared to control vector CAL‐27 cells (Figure 3A,B, *p < 0.05). In contrast, shIFIX restored β‐catenin expression to a level similar to that of normal oral mucosa (Figure 3A,B).

Next, we investigated variations in Wnt3a and Wnt4 expression upon IFIX overexpression and knockdown using qRT‐PCR and Western blotting. Since Wnt3a and Wnt4 are known to activate cell‐autonomous Wnt signalling and control stem cell activity, we found that IFIX overexpression significantly inhibited the mRNA and protein expression of Wnt3a and Wnt4 (Figure 3C, ***p < 0.001). Conversely, shIFIX increased Wnt3a and Wnt4 protein expression to levels exceeding the normal range (Figure 3C–F).

We also investigated the expression of NKD2, an intracellular canonical Wnt signalling inhibitor. NKD2 forms a mutual degradation complex with Dvl‐1, thereby inhibiting Wnt signalling by altering Wnt/Dvl/β‐catenin signalling. The results showed that IFIX upregulated NKD2 at both the mRNA and protein levels (Figure 3G,H, ***p < 0.001), while shIFIX significantly downregulated NKD2 expression (Figure 3D, **p < 0.01).

These findings suggest that IFIX inhibits β‐catenin expression by regulating key members of the Wnt signalling pathway, including Wnt3a, Wnt4 and NKD2. This regulatory effect likely inhibits the activation of the Wnt signalling pathway, thereby suppressing cell migration and invasion capabilities during the EMT process [14, 15]. Furthermore, the upregulation of NKD2 may enhance this inhibitory effect, as NKD2 is a negative regulator of the Wnt signalling pathway and inhibits Wnt/β‐catenin signalling by degrading Dvl‐1 [16]. These results indicate that the antitumour effects of IFIX in OSCC cells may be partially attributed to its inhibition of the Wnt signalling pathway, thereby preventing EMT and tumour progression. We captured the same trend in the SCC‐25 (Figure S1).

### 3.4 Silencing NKD2 Induces EMT in CAL‐27 Cells With Overexpressed or Silenced IFIX

**FIGURE 4:**
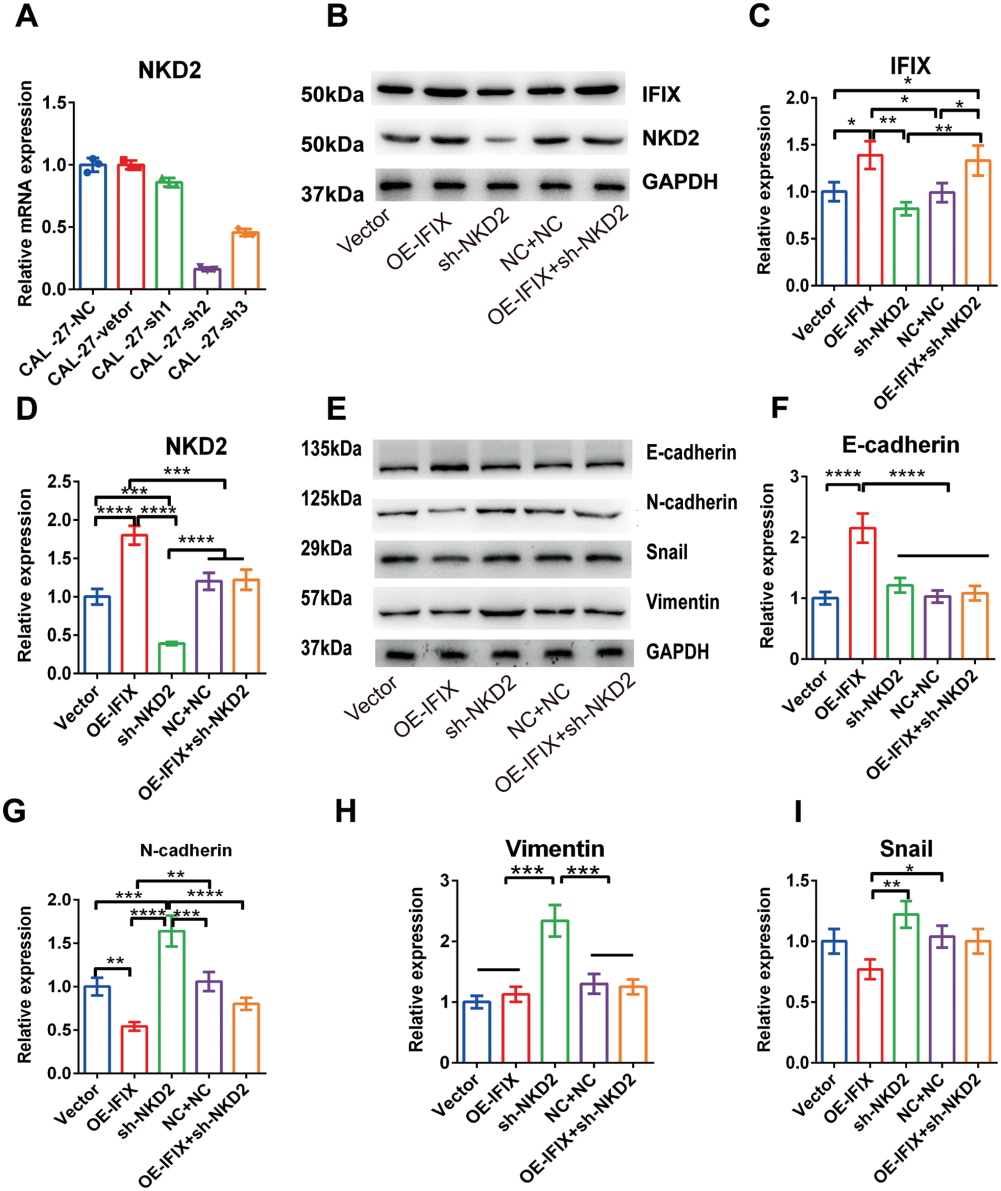
Silencing NKD2 induces EMT in CAL‐27 cells with overexpressed or silenced IFIX. (A) qRT‐PCR analysis quantifying NKD2 expression in CAL‐27 cells with stable expression of shRNA molecules. NKD2 expression levels in CAL‐27 cells with various treatments, including control, overexpressed IFIX (OE‐IFIX) and silenced IFIX (shIFIX), are shown. (B) Western blot analysis of NKD2 and IFIX protein levels in CAL‐27 cells with different treatments: vector control, OE‐IFIX, NC + NC, sh‐NKD2 and OE‐IFIX + sh‐NKD2. (C) Quantification of IFIX protein levels from Western blot analysis. (D) Quantification of NKD2 protein levels from Western blot analysis. (E) Western blot analysis of EMT markers (E‐cadherin, N‐cadherin, Snail and vimentin) in response to IFIX silencing by siRNA molecules in CAL‐27 cells with different treatments. (F‐I) Quantification of E‐cadherin, N‐cadherin, vimentin and Snail protein levels from Western blot analysis. Vector: OE‐IFIX CAL‐27‐vector cells; OE‐IFIX: overexpressed IFIX‐CAL‐27 cells; sh‐NKD2: Sh‐NKD2‐CAL‐27 cells; NC + NC: OE‐IFIX‐CAL‐27‐vector + sh‐NKD2 CAL‐27‐vector cells; OE‐IFIX + sh‐NKD2: OE‐IFIX + sh‐NKD2‐CAL‐27 cells. Error bars indicate mean ± SD. *p < 0.05, **p < 0.01, ***p < 0.001, ****p < 0.0001.

For the results of IFIX on SCC‐25 are consistent with that of CAL‐27, we investigated whether IFIX affects EMT through NKD2 in the context of Wnt signalling only in CAL‐27 cells. NKD2 expression was suppressed in IFIX CAL‐27 cells overexpressing shNKD2‐1, shNKD2‐1 or shNKD2‐3 lentivirus (Figure 4A). Subsequently, the intracellular levels of IFIX, NKD2 and EMT markers were assessed.

Off‐target effects were excluded by incubating CAL‐27 cells overexpressing IFIX with shNKD2 and CAL‐27 cells with siNKD2 for 48 h. Western blotting results confirmed that NKD2 was overexpressed in OE‐IFIX CAL‐27 cells compared to the expression in CAL‐27 cells, and that expression was flat compared to that in the controls, including vector (OE‐IFIX CAL‐27‐vector) and normal control (NC) + NC (OE‐IFIX CAL‐27‐vector + sh‐NKD2 CAL‐27‐vector) (Figure 4B,C). Western blotting also showed that NKD2 did not affect IFIX expression (Figure 4B,D).

To understand the interplay between NKD2 and EMT in CAL‐27 cancer cells regulated by IFIX, we silenced NKD2 and then rescued it by transfection with a plasmid‐overexpressing IFIX. E‐cadherin levels did not significantly change in cells incubated with shNKD2 compared with those in cells incubated with shNC + NC and the control vector (*p* < 0.0001). However, E‐cadherin levels were significantly increased in cells overexpressing IFIX. In contrast, N‐cadherin, Snail and vimentin levels were higher in cells incubated with shNKD2 than in those incubated with shNC + NC and the control vector (*p* < 0.0001; Figure 4E–I).

These results suggest that silencing NKD2 induces EMT in CAL‐27 cells, even when IFIX is overexpressed. This indicates that NKD2 plays a crucial role in mediating the effects of IFIX on EMT. Specifically, the upregulation of E‐cadherin and downregulation of N‐cadherin, Snail and vimentin in IFIX‐overexpressing cells is reversed when NKD2 is silenced, suggesting that NKD2 is a key mediator of IFIX's inhibitory effects on EMT.

### 3.5 NKD2 is a Key Mediator of the Antitumour Effect of IFIX in OSCC

**FIGURE 5:**
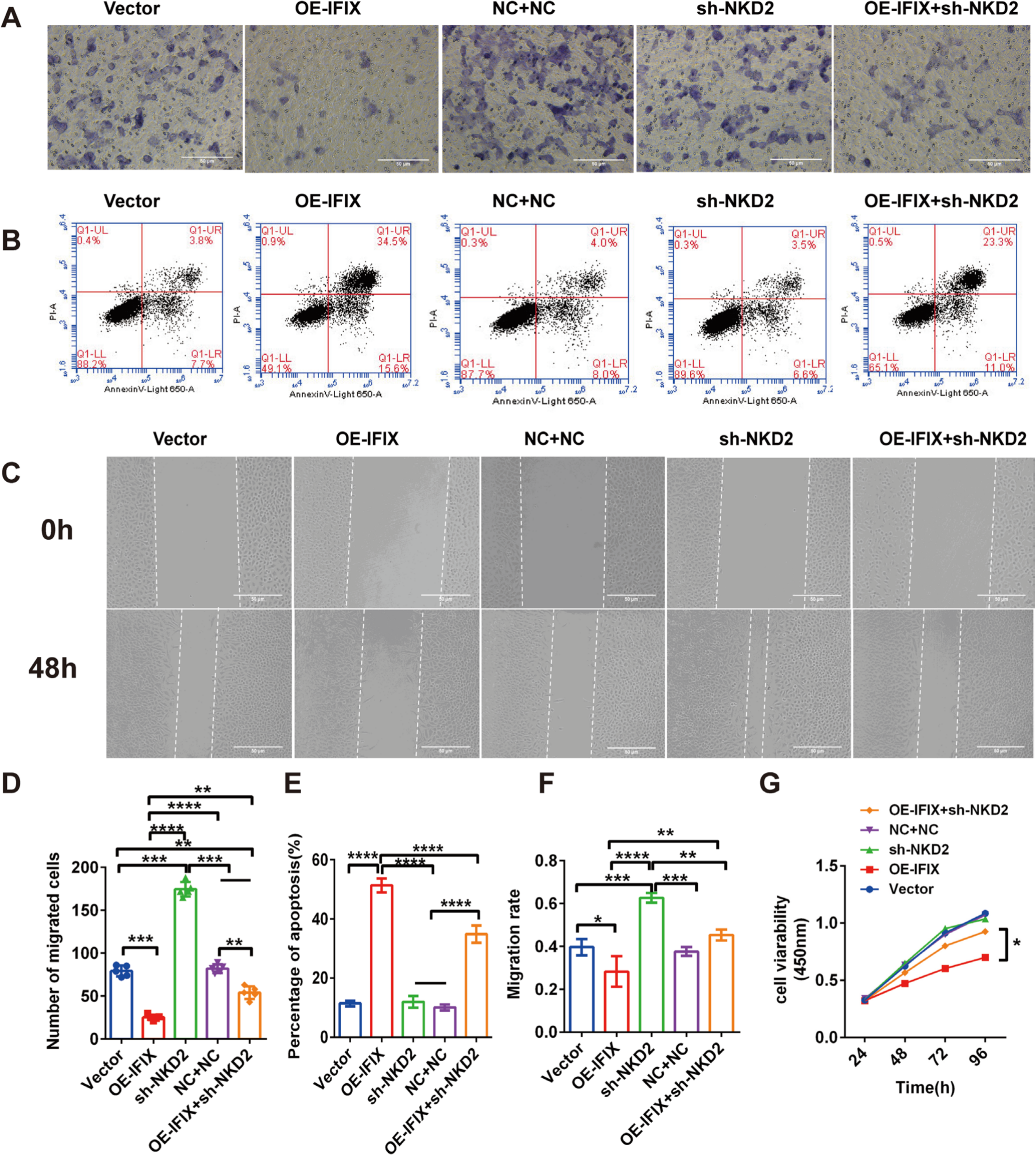
Effects of NKD2 and IFIX on OSCC cell proliferation, migration, apoptosis and EMT in vitro. (A) Transwell assay showing the invasive ability of NC + NC‐, OE‐IFIX‐, sh‐NKD2‐ and OE‐IFIX + sh‐NKD2‐treated CAL‐27 cells. Images captured at 20× magnification. (B) Flow cytometry analysis of apoptosis in NC + NC‐, OE‐IFIX‐, sh‐NKD2‐ and OE‐IFIX + sh‐NKD2‐treated CAL‐27 cells. (C) Wound healing assay showing migration capability of NC + NC‐, OE‐IFIX‐, sh‐NKD2‐ and OE‐IFIX + sh‐NKD2‐treated CAL‐27 cells at 0 h and 48 h. Images captured at 20× magnification. (D) Quantitative analysis of invasion from the Transwell assay. Error bars indicate mean ± SD. *p < 0.05, **p < 0.01, ***p < 0.001, ****p < 0.0001. (E) Quantitative analysis of apoptosis from the flow cytometry assay. Error bars indicate mean ± SD. *p < 0.05, **p < 0.01, ***p < 0.001, ****p < 0.0001. (F) Quantitative analysis of migration from the wound healing assay. Error bars indicate mean ± SD. *p < 0.05, **p < 0.01, ***p < 0.001, ****p < 0.0001. (G) CCK‐8 assay results showing cell proliferation in NC + NC‐, OE‐IFIX‐, sh‐NKD2‐ and OE‐IFIX + sh‐NKD2‐treated CAL‐27 cells. Error bars indicate mean ± SD. *p < 0.05, **p < 0.01, ***p < 0.001, ****p < 0.0001.

The increased proliferation of NKD2‐silenced cells was rescued by transfection with a plasmid‐overexpressing IFIX (*p* < 0.0001). Cell proliferation, invasion, migration and apoptosis were assessed using Transwell, flow cytometric assays, wound healing and the CCK‐8 assay, respectively (*p* < 0.0001; Figure 5A–F).

To confirm the role of NKD2 in the context of IFIX, we evaluated the migration and invasion of OE‐IFIX CAL‐27 cells transfected with sh‐NKD2, shNC + NC or control vectors. Transwell and wound healing assays demonstrated that shNKD2 transfection significantly enhanced migration, invasion and motility of CAL‐27 cells compared to the shNC + NC and control vector groups (*p* < 0.0001; Figure 5A,C–E). Additionally, flow cytometry showed a significant decrease in apoptosis in NKD2‐silenced cells, whereas IFIX overexpression markedly increased apoptosis (*p* < 0.0001; Figure 5B,E). The CCK‐8 assay further confirmed that IFIX overexpression suppressed cell proliferation, while NKD2 silencing promoted it (*p* < 0.0001; Figure 5G). These findings support the hypothesis that NKD2 inhibits cancer cell growth in vitro.

**FIGURE 6:**
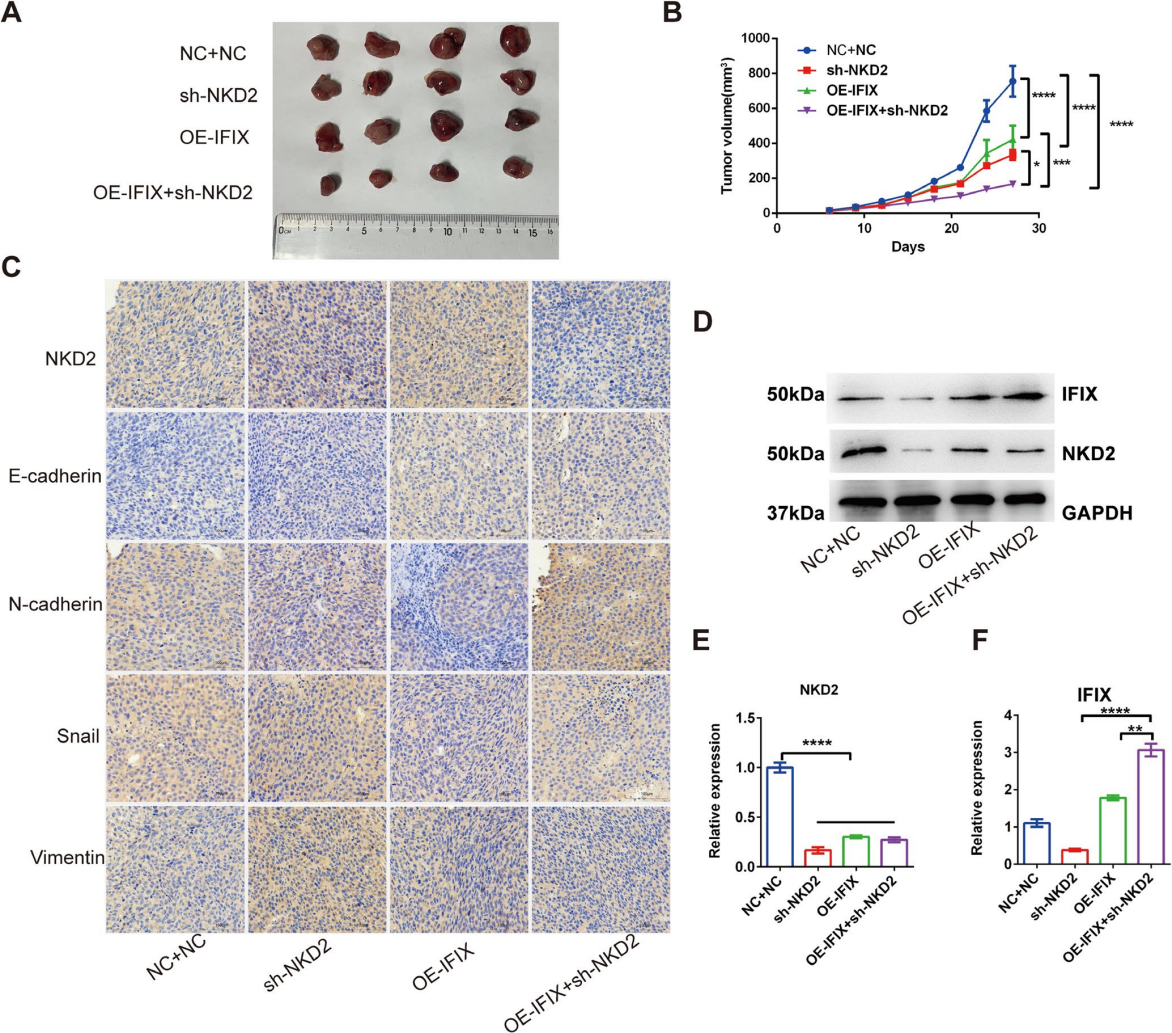
Effects of NKD2 and IFIX on tumour growth and EMT in vivo. (A) Tumour growth curves of SCC‐25 cells with stable expression of sh‐NKD2, OE‐IFIX, OE‐IFIX + sh‐NKD2 or NC + NC grafted into nude mice (*n* = 4). Tumour volume was measured over time. Error bars indicate mean ± SD. *p < 0.05, **p < 0.01, ***p < 0.001, ****p < 0.0001. (B) Images of tumours excised from nude mice at the end of the experiment. (C) Immunohistochemistry (IHC) analysis of EMT markers (E‐cadherin, N‐cadherin, vimentin and Snail) and NKD2 expression in tumour tissues from NC + NC, sh‐NKD2, OE‐IFIX and OE‐IFIX + sh‐NKD2 groups. Images captured at 20× magnification. (D) Western blot analysis of IFIX and NKD2 protein levels in tumour tissues, with GAPDH as a loading control. (E) Quantification of tumour weights from excised tumours. Error bars indicate mean ± SD. *p < 0.05, **p < 0.01, ***p < 0.001, ****p < 0.0001. (F) Quantification of relative protein levels of IFIX and NKD2 from Western blot analysis. Error bars indicate mean ± SD. *p < 0.05, **p < 0.01, ***p < 0.001, ****p < 0.0001.

Given the modulation of NKD2 by IFIX observed in vitro (Figure 6A–F), we further examined these effects in vivo using a mouse xenograft model. SCC‐25 cells with sh‐NKD2, OE‐IFIX, OE‐IFIX+Sh‐NKD2 and control (NC + NC) were subcutaneously injected into nude mice. Tumour growth was significantly enhanced in the NC + NC group compared to all other groups (*p* < 0.0001; Figure 6A,B). Notably, no significant difference in tumour volume was observed between the Sh‐NKD2 and OE‐IFIX groups, indicating that NKD2 knockdown and IFIX overexpression have comparable effects on tumour growth suppression. Importantly, the OE‐IFIX+Sh‐NKD2 group exhibited the smallest tumour volume, with significant differences compared to all other groups, including OE‐IFIX and Sh‐NKD2 (*p* < 0.0001). This result suggests that IFIX retains its antitumour effect even in the absence of NKD2.

Western blot analysis demonstrated upregulation of NKD2 and IFIX in the OE‐IFIX and OE‐IFIX+Sh‐NKD2 groups, consistent with their tumour‐suppressive effects. As expected, NKD2 expression was significantly reduced in the Sh‐NKD2 and OE‐IFIX+Sh‐NKD2 groups due to knockdown (Figure 6D–F).

To further investigate the roles of IFIX and NKD2 in EMT regulation, immunohistochemical staining (IHC) was performed to assess the expression of NKD2 and EMT markers (E‐cadherin, N‐cadherin, vimentin and Snail) in the xenograft tumours (Figure 6C). NKD2 expression was significantly reduced in the Sh‐NKD2 group compared to the NC + NC group, confirming effective knockdown of NKD2. In contrast, NKD2 expression was markedly upregulated in the OE‐IFIX group, indicating that IFIX overexpression enhances NKD2 expression. As expected, NKD2 expression was low in the OE‐IFIX+Sh‐NKD2 group due to knockdown but remained partially detectable.

E‐cadherin, an epithelial marker, was significantly increased in the OE‐IFIX and OE‐IFIX+Sh‐NKD2 groups compared to the NC + NC and Sh‐NKD2 groups, suggesting strong EMT inhibition by IFIX. Notably, E‐cadherin expression was highest in the OE‐IFIX+Sh‐NKD2 group, indicating that IFIX retains its EMT‐suppressive effects even in the absence of NKD2. Conversely, the mesenchymal markers N‐cadherin, vimentin and Snail were highly expressed in the NC + NC and Sh‐NKD2 groups, reflecting EMT activation. These markers were slight downregulated in the OE‐IFIX group and OE‐IFIX+Sh‐NKD2 group except N‐cadherin, demonstrating that IFIX inhibits EMT.

These results indicate that NKD2 is a critical mediator of the antitumour effects of IFIX in OSCC. By modulating NKD2, IFIX exerts significant control over cell proliferation, migration, invasion and EMT, underscoring its potential role in tumour suppression. The integration of in vitro and in vivo data, along with IHC findings, supports the notion that targeting the IFIX‐NKD2 axis could be a promising therapeutic strategy for OSCC.

### 3.6 Effects of IFIX on Lymph Node Metastasis

**FIGURE 7:**
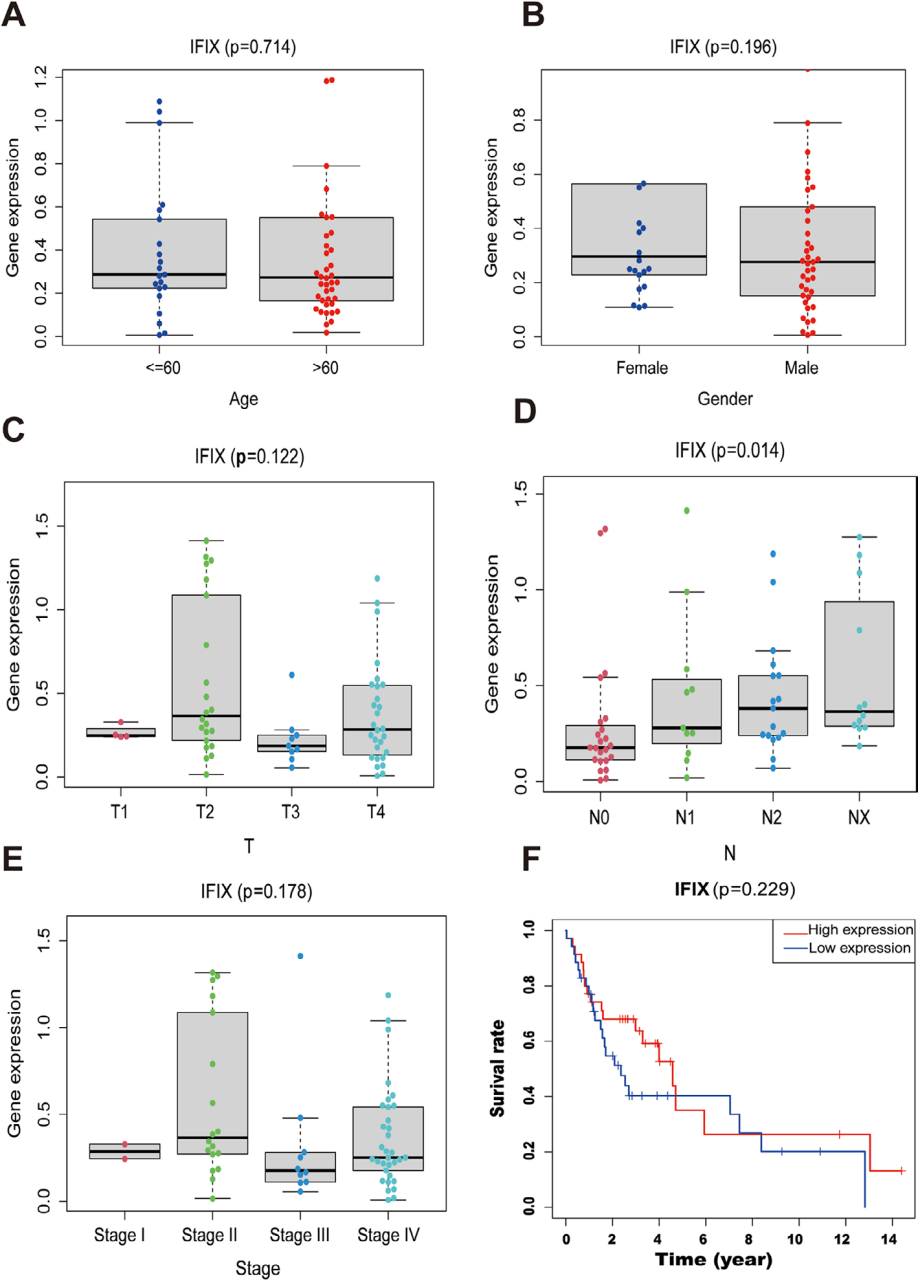
Correlation between IFIX expression and clinicopathological parameters in OSCC. (A) Box plot showing the correlation between IFIX expression and age (< 60 and ≥ 60 years). The p‐value is 0.714, indicating no significant correlation. (B) Box plot showing the correlation between IFIX expression and gender (female and male). The p‐value is 0.198, indicating no significant correlation. (C) Box plot showing the correlation between IFIX expression and tumour stage (T1, T2, T3 and T4). The p‐value is 0.122, indicating no significant correlation. (D) Box plot showing the correlation between IFIX expression and nodal metastasis (N0, N1, N2 and NX). The *p*‐value is 0.014, indicating a significant correlation. (E) Box plot showing the correlation between IFIX expression and cancer stage (Stage I–III and IV). The *p*‐value is 0.178, indicating no significant correlation. (F) Kaplan–Meier survival curve comparing overall survival rates of OSCC patients with high and low IFIX expression levels. The *p*‐value is 0.229, indicating no significant difference in survival rates between the two groups.

Additionally, we examined the correlation between IFIX expression and overall survival (OS) in patients with oral cancer using TCGA data (Figure 7A–F). Although there was a trend towards improved survival with increased IFIX expression, the results did not reach statistical significance (*p* = 0.229; Figure 7F). These findings suggest that while IFIX expression is linked to reduced lymph node metastasis, it may not be a standalone predictor of overall prognosis (Figure 7D).

The association between IFIX expression and reduced lymph node metastasis underscores the potential role of IFIX in inhibiting EMT and metastatic spread in OSCC. However, the lack of a significant impact on OS suggests that other factors and pathways may also contribute to patient outcomes. Further studies are needed to elucidate these mechanisms and to determine the potential of IFIX as a therapeutic target in OSCC.

## 4 Discussion

We investigated the potential biological mechanism through which IFIX regulates the EMT process in oral cancer in vitro. The Wnt signalling pathway is likely a key factor in mediating EMT in the context of IFIX expression. Signalling by the Wnt family is a central mechanism underlying the regulation of tissue morphogenesis during embryogenesis and repair [4, 5, 6, 7, 8]. The pivot of this signalling cascade is the Wnt ligand, which binds to receptors in the Frizzled family or the ROR1/ROR2 and RYK families [8]. This interaction governs downstream canonical and noncanonical signalling pathways, including Wnt PCP, ultimately influencing the cytoskeleton, transcriptional control of proliferation and differentiation, and organelle dynamics. Canonical Wnt signalling is transduced through Frizzled receptors and the LRP5/LRP6 coreceptor to activate the β‐catenin signalling cascade [8].

**FIGURE 8:**
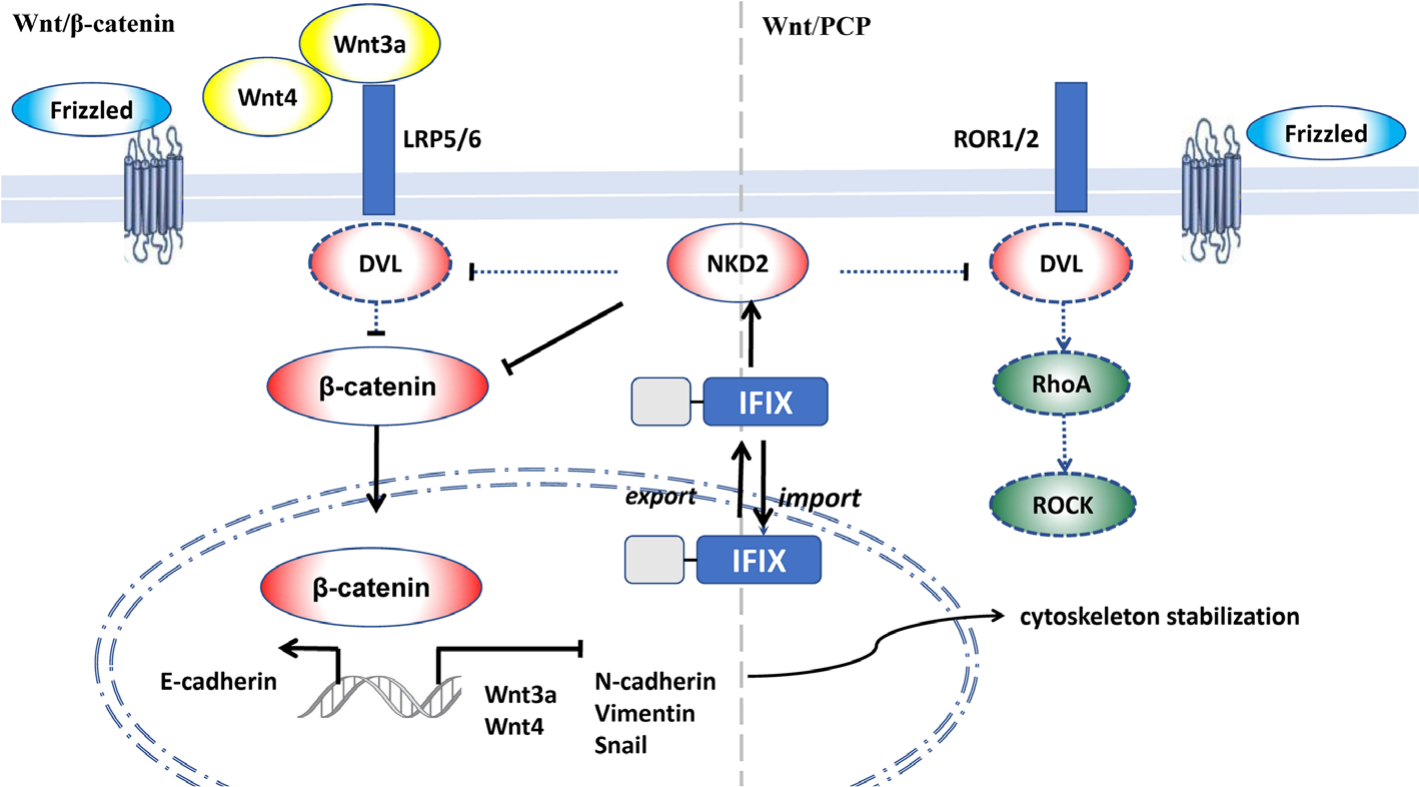
Schematic representation of IFIX regulation of the Wnt signalling pathways in OSCC. IFIX inhibits β‐catenin, affecting the canonical pathway and the expression of EMT markers. Dashed lines indicate hypothetical pathways that have not yet been experimentally confirmed. This study has demonstrated the role of IFIX in regulating NKD2 expression and its downstream effects on Wnt signalling and EMT, but further research is needed to validate these proposed interactions. *Note:* The roles of Frizzled, DVL and LRP5/6 in the diagram are based on established Wnt signalling pathways but were not directly investigated in this study. The hypothetical interactions (dashed lines) represent areas for future exploration to fully elucidate the mechanistic pathways involved.

We investigated β‐catenin expression in OSCC cells with abundant or repressed IFIX expression in normal oral mucosa cells by analysing the expression of Wnt4 and Wnt3a, which activate cell‐autonomous Wnt signalling independently of porcupine O‐acyltransferase or Wnt secretion [17]. During biological development, Wnt3a promotes embryonic axis duplication, whereas Wnt4 is involved in morphogenetic movements restricting the embryonic axis [17]. Importantly, Wnt3a and Wnt4 are significantly associated with EMT in tumorigenesis. Overexpressed Wnt3a regulates EMT and the progression of several malignant cancers, including OSCC [18, 19, 20]. Wnt4 regulates focal adhesion kinase activation and promotes cell movement in Drosophila during ovarian morphogenesis [21]. We previously showed that IFIX inhibits paxillin, which mediates adhesion disassembly at the cell front via the focal adhesion kinase/Src complex [2]. These findings suggest that IFIX inhibits EMT through the Wnt signalling pathway (Figure 8). We also found that IFIX restricts Wnt3a and Wnt4 expression. These findings suggest that the antitumour effect of IFIX is significantly associated with canonical and noncanonical Wnt signalling pathways. However, the precise pathway through which IFIX regulates Wnt3a and Wnt4 remains unclear (Figure 8).

To further explore the inhibition of Wnt signalling by IFIX, we analysed NKD2, an inhibitor of the Wnt signalling pathway. NKD2 negatively regulates Wnt receptor signalling via interaction with dishevelled family proteins and escorts exocytic vesicles containing transforming growth factor‐alpha to the basolateral membrane of polarised epithelial cells [22]. Many malignant cancers are suppressed through the NKD2‐mediated inhibition of Wnt signalling [5]. Our results show that IFIX restricts Wnt signalling by increasing NKD2 expression, consequently reversing the EMT process. Silencing NKD2 significantly increased OSCC cell proliferation, invasion and migration in vitro (Figures 5, and 6). Interestingly, while in vitro experiments demonstrated that NKD2 knockdown significantly promoted EMT and tumour cell proliferation, the in vivo xenograft model revealed no significant difference in tumour volume between the Sh‐NKD2 and OE‐IFIX groups. However, the OE‐IFIX+Sh‐NKD2 group exhibited the smallest tumour volume, suggesting that IFIX retains its antitumour effects even in the absence of NKD2. This discrepancy highlights the complexity of tumour biology in vivo, which is influenced by factors such as the tumour microenvironment, immune regulation and metabolic adaptation [23, 24]. These findings imply that IFIX may exert its effects through NKD2‐independent pathways in vivo, warranting further investigation.

While our study provides valuable insights, it has certain limitations. First, the discrepancy between in vitro and in vivo results underscores the influence of the tumour microenvironment and immune response in vivo. Second, the use of a single cell line and xenograft model may not fully capture the heterogeneity of OSCC [25]. Third, although we demonstrated Wnt signalling and EMT involvement, the precise mechanisms by which IFIX regulates these pathways remain unclear. Lastly, the clinical relevance of IFIX as a prognostic marker requires validation in larger patient cohorts. Addressing these limitations in future studies will strengthen the translational potential of our findings.

It has been previously demonstrated that IFIX inhibits OSCC cell biological behaviour in vitro. Furthermore, TCGA database contains much information about human OSCC. We found that IFIX was significantly associated with regional lymph nodes. Previous findings suggest that EMT is important for metastasis, although whether it is essential or not for this process remains obscure [26]. EMT is considered the initial step in the invasion–metastasis cascade of OSCC [27]. However, the OS rate associated with IFIX anticancer effects was not statistically significant.

In the present study, the clinical relevance of IFIX expression was not statistically significant in terms of sex, age, TNM, clinical stages and OS rates at 14 years. Significantly fewer regional lymph nodes more abundantly expressed IFIX in nonlymph node metastasis (*p* = 0.014). An evaluation of clinicopathological parameters and IFIX expression revealed that the OS rate of patients with increased IFIX expression was optimistic at over 100 months of follow‐up, although the *p*‐value was 0.056 [28]. However, the improved OS was more obvious at 168 months. Identification of the risk factors and predictive biomarkers of recurrence is imperative. Although Ding et al. evaluated prognoses and biological mechanisms, a single protein marker has yet to be validated for clinical applications [28]. The inhibitory role of IFIX in OSCC is complicated and involves EMT and other biological processes, such as cancer stem cells (CSCs) and tumour metabolism associated with mitochondria.

Tumour cells might undergo the phenotypic changes that comprise EMT. An unusual combination of EMT and stem cell competence might result in the formation of CSCs. Canonical Wnt signals are involved in stem cell signalling networks [5]. The balance between Wnt‐fibroblast growth factor‐Notch and bone morphogenetic protein‐Hedgehog signalling networks is important for maintaining homeostasis between stem and progenitor cells [4]. Nevertheless, data about Wnt and CSCs are unclear, possibly because an epigenetic mechanism is involved in IFIX, CSC markers and the tumour environment. Further studies are needed for verification.

Our findings suggest that IFIX, through its regulation of NKD2 and EMT, holds potential as a prognostic marker and therapeutic target for OSCC [29, 30]. IFIX's ability to modulate EMT and Wnt signalling pathways underscores its broader role in tumour suppression and metastasis prevention. Future studies should explore the translational applications of IFIX, including its use in targeted therapies and combination treatments to enhance antitumour effects [31]. Moreover, elucidating the upstream regulators and downstream effectors of IFIX may reveal novel therapeutic strategies for OSCC and other malignancies.

## Author Contributions

**Shan Wang:** conceptualization (equal), data curation (equal), formal analysis (equal), funding acquisition (equal), investigation (equal), methodology (equal), project administration (equal), resources (equal), software (equal), supervision (equal), validation (equal), visualization (equal), writing – original draft (equal), writing – review and editing (equal). **Haixia Fan:** investigation (equal), methodology (equal), resources (equal). **Jie Bai:** writing – review and editing (equal).

## Ethics Statement

The research protocol was approved by the Hainan Medical University Institutional Reviewer Board.

Informed Consent: N/A.

Registry and the Registration No: N/A.

Animal Studies: The research protocol was approved by the Hainan Medical University Institutional Reviewer Board.

## Consent

All authors agreed on the manuscript.

## Conflicts of Interest

The authors declare no conflicts of interest.

## Supporting information


**Figure S1.** Regulation of Wnt signalling components by IFIX in OSCC‐25 cells.
Western blot analysis shows the expression levels of β‐catenin, Wnt3a, Wnt4 and NKD2 in different treatment groups, including normal mucosa, SCC‐25‐vector, SCC‐25NC and SCC‐25 cells overexpressing IFIX (SCC‐25‐OE), and SCC‐25 cells with IFIX knockdown (SCC‐25‐sh). GAPDH is used as a loading control. Quantification expression levels from Western blot results. Error bars represent mean ± SD. *p < 0.05, **p < 0.01, and ***p < 0.001.

## Data Availability

The data supporting the findings of this study are available from the corresponding author upon reasonable request.
