# Supplementary figures and images for: NKD2 as a Mediator of IFIX Antioncogene‐Induced Wnt Signalling and Epithelial–Mesenchymal Transition in Human OSCC
Source: J Cell Mol Med. 2025 Jan 20;29(2):e70342. doi: 10.1111/jcmm.70342 (PMC11745820; doi:10.1111/jcmm.70342)

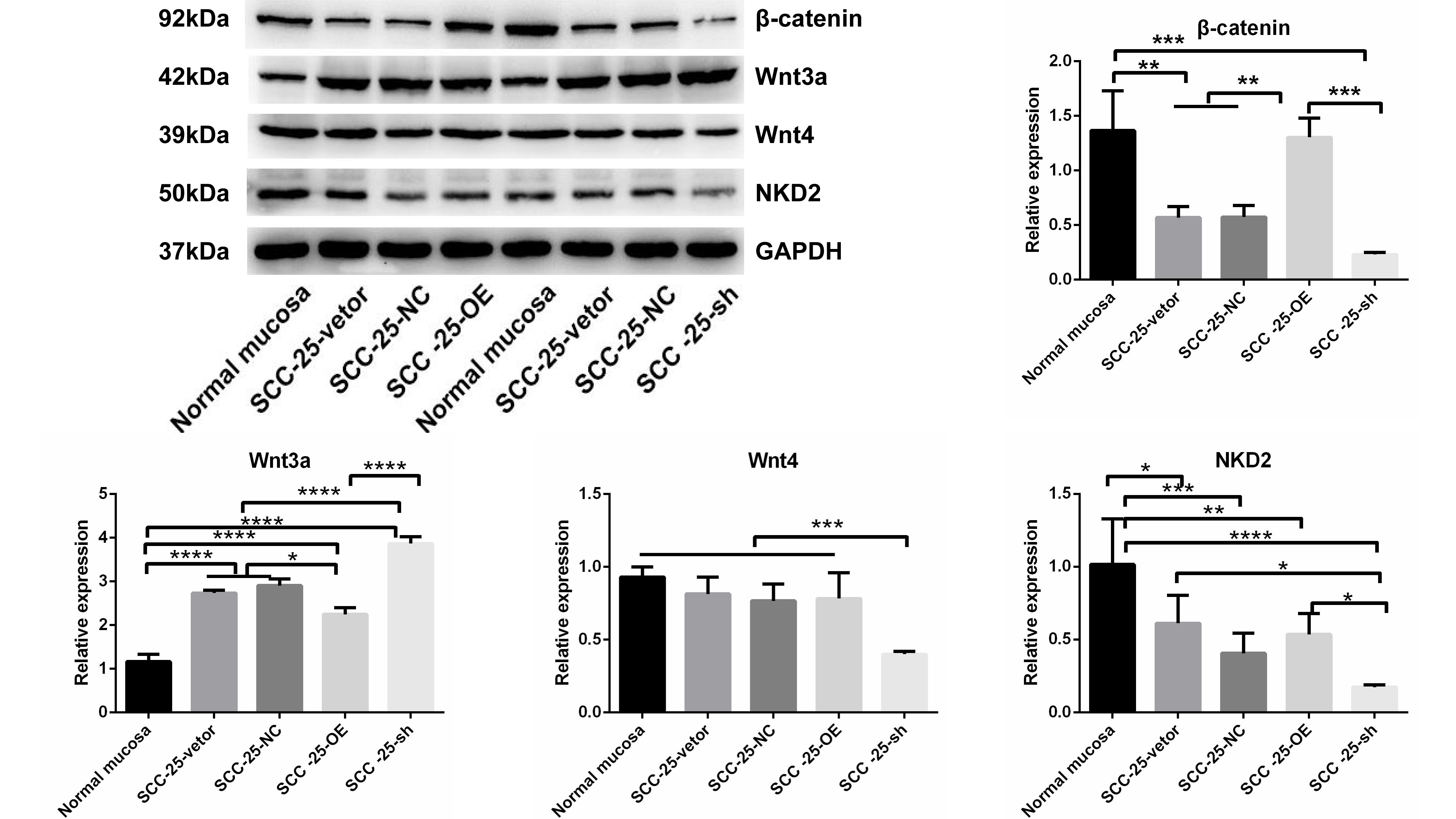

Supplement: Supplementary file 1 — Figure S1. Regulation of Wnt signalling components by IFIX in OSCC‐25 cells. Western blot analysis shows the expression levels of β‐catenin, Wnt3a, Wnt4 and NKD2 in different treatment groups, including normal mucosa, SCC‐25‐vector, SCC‐25NC and SCC‐25 cells overexpressing IFIX (SCC‐25‐OE), and SCC‐25 cells with IFIX knockdown (SCC‐25‐sh). GAPDH is used as a loading control. Quantification expression levels from Western blot results. Error bars represent mean ± SD. *p < 0.05, **p < 0.01, and ***p < 0.001. [file JCMM-29-e70342-s001.tif]
